# Supplementary material for: A metagenomics method for the quantitative detection of bacterial pathogens causing hospital-associated and ventilator-associated pneumonia
Source: Microbiol Spectr. 2023 Oct 27;11(6):e01294-23. doi: 10.1128/spectrum.01294-23 (PMC10715005; doi:10.1128/spectrum.01294-23)
Supplement: Supplemental material — Additional method information with supporting figures and tables. [file spectrum.01294-23-s0001.docx]

Additional File

## Metagenomics methods for the quantitative detection of pathogens causing ventilator-associated pneumonia

# Content

[Additional method information 1](#_Toc84406236)

[Accuracy of SPC detection 1](#_Toc84406237)

[Detailed workflow for sequence data analysis and interpretation 2](#_Toc84406238)

[Normalization of sequence read counts 2](#_Toc84406239)

[SOI and SPC detection 2](#_Toc84406240)

[Calculation of absolute concentration of detected SOI 4](#_Toc84406241)

[Calculation of minimal detectable concentration of SOI 5](#_Toc84406242)

[Determination of metagenomics threshold 6](#_Toc84406243)

[Additional figures 6](#_Toc84406244)

[Additional Tables 10](#_Toc84406245)

[Additional references 12](#_Toc84406246)

# Additional method information

## Accuracy of SPC detection

We assessed the accuracy of taxonomic classification of sequence reads derived from SPC and their potential confusion with SOI(s) by Kraken V0.10.5b [1], a tool used in our bioinformatics pipeline (see Additional figure 1). The measured precision is above 99.95 % with a sensitivity of 98.7 %. These figures show that the sequence reads of the SPC can be efficiently distinguished from those of SOIs, bacterial flora and patient DNA.

## Detailed workflow for sequence data analysis and interpretation

The complete workflow for quantitative detection of SOI by mNGS is presented in Figure 1 in the main text. The following paragraphs provide additional details on data interpretation applied to each sample after the demultiplexing step and taxonomic classification of sequences. All SOIs were analyzed separately as independent detection assays.

### Normalization of sequence read counts

Given that the total number of reads and the proportion of human reads vary between sequencing runs, a normalization step is needed for comparing results from different samples. We normalized the numbers of sequence reads classified to a bacterial species of interest (*R_SOI_*) or to the SPC (*R_SPC_*) with the total number of reads associated to all bacterial species (SOI, SPC and other species from the database) (*R_bacteria_*). The normalized values (*RN_SOI_* and *RN_SPC_*) were expressed in reads per million of bacterial reads (RPMB):

${RN}_{SOI}=\frac{R_{SOI}}{R_{bacteria}}\times{10}^{6}$ and ${RN}_{SPC}=\frac{R_{SPC}}{R_{bacteria}}\times{10}^{6}$

### SOI and SPC detection

Correct taxonomic classification of SOIs and SPC sequences is key to mNGS-based diagnostic, to prevent false positive or false negative detections. Taxonomic classification errors can arise for several reasons such as sequencing errors, inaccuracy of classification algorithm or low quality of genomic sequences within the reference database [2]. Moreover, the presence of contaminating DNA in the reagents may also induce false detection of species [3, 4].

Generally, a negative control is analyzed by sequencing a buffer that would follow the same workflow as the sample. However, the DNA extracted from the negative control is too low for efficient sequencing assay. In addition, BAL samples, even when negative in microbial culture, are not sterile and may contain a flora composed by many species at concentrations that can exceed 10^3^ CFU/mL. We have therefore made a different technical choice by developing a detection threshold (DT) for each species to differentiate background from ‘true’ taxonomic assignments of sequence reads to SPC or SOI. *DT_SPC_* and *DT_SOI_* represent the minimal count of classified sequence reads normalized per million of bacterial reads required to report SPC or SOI as detected.

#### Definition of DT_SPC_

To evaluate the possibility for distinguishing the natural presence of *B. subtilis* in BAL sample from spiked SPC and to define *DT_SPC_*, we compared the quantity of sequence reads classified as *B. subtilis* on 7 non-spiked and on 45 SPC-spiked BAL samples. The median normalized quantity of sequences classified as *B. subtilis* in non-spiked samples was 5 RPMB and 12,000 RPMB in SPC-spiked samples (Additional figure 2). This result reveals the low level of misclassification of sequences to *B. subtilis* and/or the presence of *B. subtilis* in BAL samples or in reagents, as a contaminant, in amounts substantially lower than the one which is spiked as SPC. We calculated, with the 3 sigma method (mean plus 3 standard deviations), a detection threshold (*DT_SPC_*) at 475 RPMB. In 40 of 45 spiked samples (88.9 %) from the “training set”, normalized quantities of sequence reads classified as *B. subtilis* were above *DT_SPC_*. The remaining 5 samples, in which sequence reads assigned as *B. subtilis* were below *DT_SPC_*, contained at least one SOI represented by more than 500,000 RPMB (see T17, T26, T35, T39 and T43 in Additional table 1). Most of these SOIs were also detected at high concentrations (≥ 10^5^ CFU/mL) by microbial cultures. The quantity of reads associated to all SOI in these samples were above 887,000 RPMB, leaving little room for detection of other species, including *B*. *subtilis* (SPC).

#### Definition of DT_SOI_

For each of the SOIs, we measured the background of the taxonomic classification using the samples from the “training set” that were negative in microbial culture for the assessed species. When large amounts of sequences in a tested sample were classified as an SOI that was not identified by culture, k-mer based sequence read classification (Kraken) was controlled using BLAST-based 16S/MetaPhlAn2 markers search. Confirmation of k-mer based classification by marker search may point to the mNGS detection of dead or non-growing SOIs, resulting from antibiotic treatments, or to substantial reagent contamination [3]. Therefore, the results of these samples were removed for the estimation of the background level of sequence classification (see values in italic in column “RN_SOI_” in Additional table 1). Although background sequence classifications varied between SOIs, in most cases they were below 10,000 RPMB. (see Additional figure 3).

Background taxonomic classification to *E. coli* measured up to 7,797 RPMB may reflect reagent contaminations that is documented to cause false detections by both PCR and sequencing methods [5]. In three (T16, T21 and T29) of 42 samples that remained culture-negative for *E*. *coli*, k-mer based taxonomic classification of sequence reads to *E. coli* was confirmed by MetaPhlAn2 and 16S markers search; the corresponding sequencing data were removed from the analysis of background taxonomic assignments to *E. coli*. For *Enterobacter cloacae*, the level of background sequence association was much stronger as it frequently exceeded 100,000 RPMB. After 16S/MetaPhlAn2 marker confirmation, the results of 2 out of 45 samples that were culture-negative to *E. cloacae* were removed from background taxonomic assessment. Therefore, a bulk of assignments to *E*. *cloacae* could be due to the presence of poor quality or wrongly annotated genomic sequences in the database used by Kraken for taxonomic classification.

As the observed level of background taxonomic classification varied between species (see Additional figure 3), we calculated *DT* for each SOI (*DT_SOI_*). The calculated *DT_E. cloacae_* was above 1,000,000 RPMB, making impossible the mNGS analysis for this species.

An SOI was reported as detected in the sample when normalized quantity of its reads (*RN_SOI_*) were equal or above the corresponding detection threshold (*DT_SOI_*) (see Figure 1➀&➁ in main text).

### Calculation of absolute concentration of detected SOI

In mNGS assays, relative quantity of sequence reads associated to a species is proportional to the relative DNA quantity of this species in the DNA extract [6]. As SPC is added at a defined concentration (*C_SPC_* = 1.7 x 10^4^ CFU/mL) within each sample, when both SOI and SPC are detected (Figure 1➀ in main text), SPC can be used as a calibrator to calculate the absolute concentration of detected SOI (*C_SOI_*) using the following formula:

$$C_{SOI}=\frac{R_{SOI}}{R_{SPC}}\times\frac{L_{SPC}}{L_{SOI}}\times C_{SPC}\times{Eff}_{SOI}$$

*R_SOI_* and *R_SPC_* correspond to the quantity of sequence reads associated to SOI and SPC, respectively. *L_SOI_* and *L_SPC_* correspond to the genome size of SOI and SPC, respectively. As SOI and SPC may have different DNA extraction yields, library preparation and sequencing efficiencies, an *Eff_SOI_* factor should be used to compensate differences in the mNGS process efficiency. The *Eff_SOI_* factor value can be determined empirically, for example, by comparing the bacterial concentration quantified by mNGS to the quantity of bacteria added in a pool of BAL samples. In our study we did not have enough material to determine *Eff_SOI_* for each SOI. Therefore, we used a default value of *Eff_SOI_* = 1. Since *B. subtilis* used as SPC has a genome size of 4.13 Mbp and was spiked into samples at 1.7x10^4^ GEq/mL, *C_SOI_* can be calculated using a simplified formula:

$$C_{SOI}=\frac{R_{SOI}\times7.02\times{10}^{4}}{R_{SPC}\times L_{SOI}}$$

### Calculation of minimal detectable concentration of SOI

When an SOI is not detected (Figure 1➂&➃ in main text), the assessment of its minimal detectable concentration (*Cmin_SOI_*) is important. *Cmin_SOI_* is the concentration of SOI required to generate at least the number of reads sufficient for its detection defined by *DT_SOI_*. It is calculated with the following formula:

$${Cmin}_{SOI}= \frac{{DT}_{SOI}}{{RN}_{SPC}} \times\frac{L_{SPC}}{L_{SOI}} \times C_{SPC}\times{Eff}_{SOI}$$

which, in our study, can be simplified to:

$${Cmin}_{SOI}=\frac{{DT}_{SOI}\times7.02\times{10}^{4}}{{RN}_{SPC}\times L_{SOI}}$$

### Determination of metagenomics threshold

A metagenomics threshold (*MT*) was defined as the concentration of genomes above which infection by the pathogen can be suspected. When an SOI is detected and quantified (Figure 1➀ in main text), its absolute concentration is compared to *MT* to determine whether to report a colonization or a suspected infection by the SOI.

To differentiate the populations of SOI(s) detected by culture at or above the clinical threshold from SOI(s) detected below this threshold, we calculated the *MT*. The training set consisted of 45 (mini-)BAL samples in which we assessed 19 SOIs (855 detections in total). We observed 20 SOI detections above the clinical threshold, 6 SOI detections below the clinical threshold and 829 negative detections (Additional table 1). As the determination of an *MT* for each species would require testing substantially more samples than we had at our disposal, we defined a unique *MT* for all the species composing our study panel. Genomic DNA concentrations of detected SOI were assessed by the receiver operating characteristics method [7] to differentiate between their detections above or below the clinical threshold by microbial culture (see Additional figure 4). The *MT* calculated with Youden’s Index [8] was 5.3x10^3^ GEq/mL (sensitivity: 95 %; specificity: 96. 1%).

# Additional figures


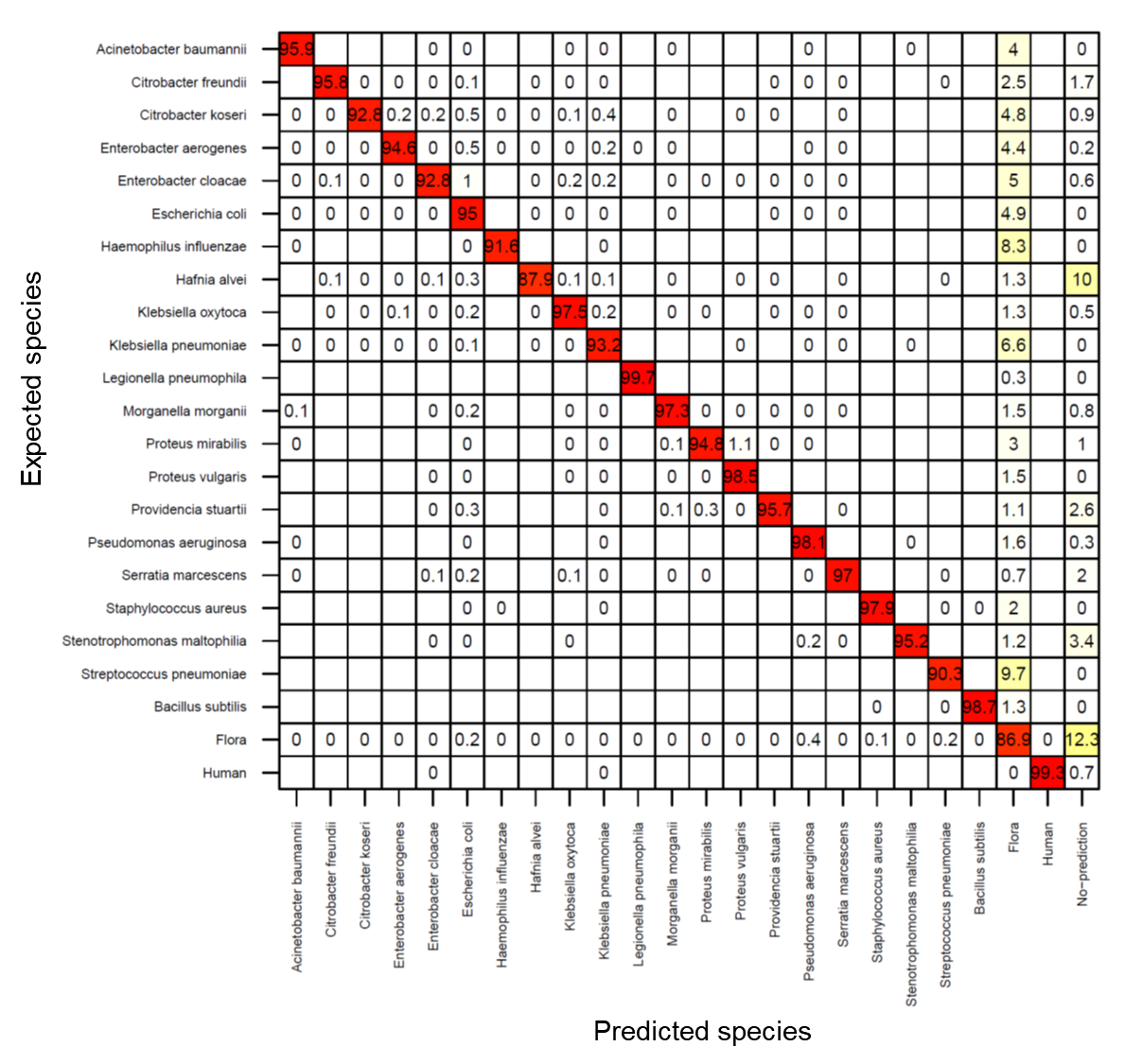


Additional figure 1: Accuracy of Kraken taxonomic binning of simulated sequence reads. Illumina 300bp paired-end reads were simulated using an in-house simulator. For each species, reads were simulated using a genome not included in the reference database. The rows correspond to the expected species (species from which the tested sequences originate) and columns to the predicted species (the taxonomic classification obtained with KRAKEN). “No-prediction” means that the read pair were not classified. The values in the boxes correspond to the sensitivity of the taxonomic classification (i.e. the percentage of reads classified to a given species) with color intensity, from red (100 %) to white (0 %). The precision of taxonomic classification of a given species is calculated as 100 % minus the sum of sensitivities obtained for other species.


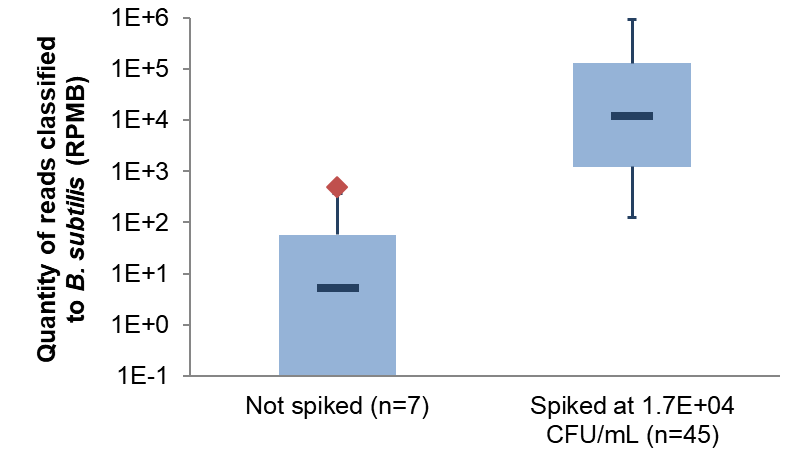


Additional figure 2: Quantity of sequence reads classified as *B. subtilis* in spiked and non-spiked BAL samples. Center lines show the medians; box limits indicate the 25th and 75th percentiles; whiskers extend to minimum and maximum values. The red diamond corresponds to the calculated *DT_SPC_* (475 RPMB).


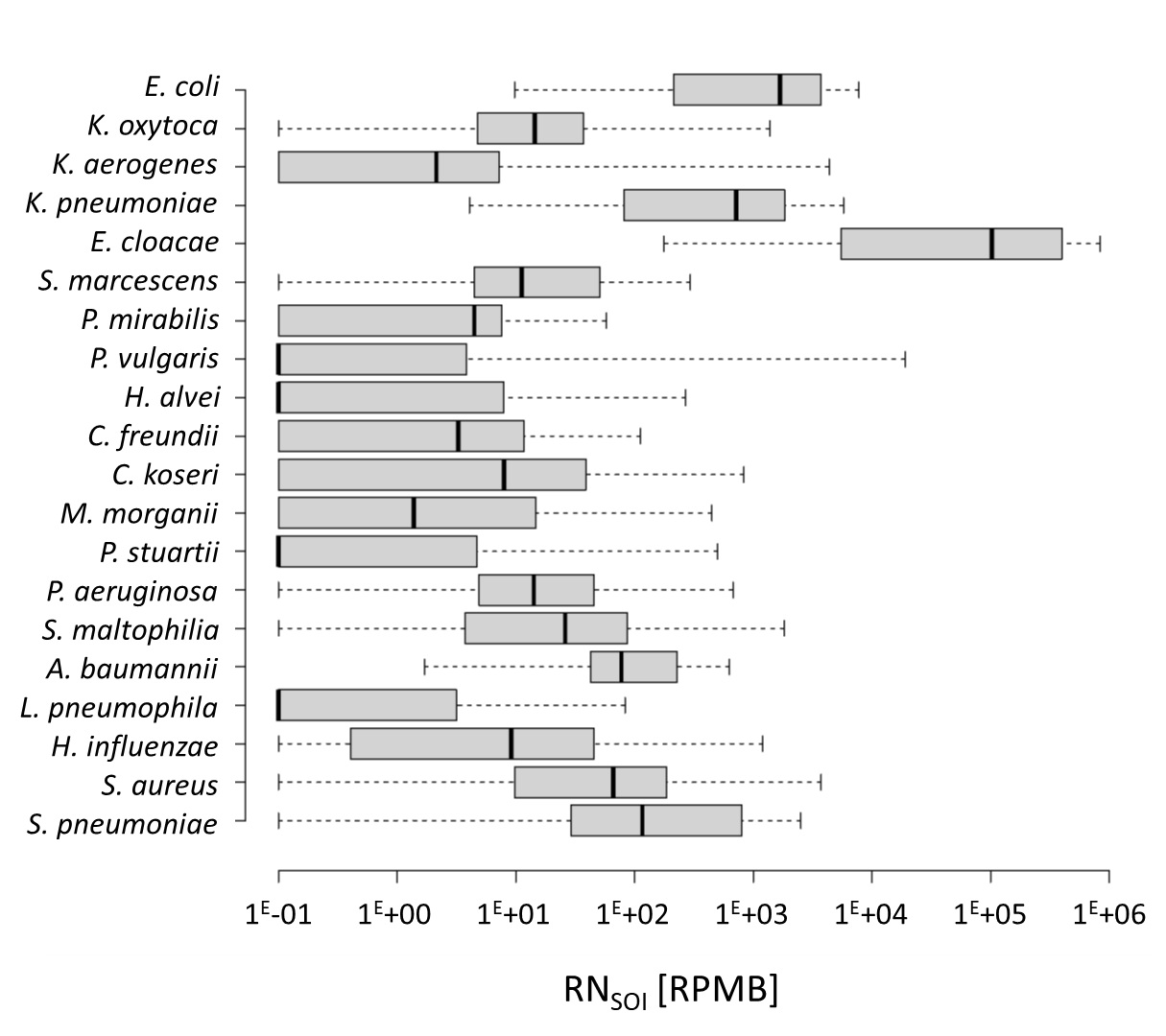


Additional figure 3: Distribution of normalized quantity of reads (*RN_SOI_*) associated to SOI in samples culture negative for tested SOI. Center lines show the medians; box limits indicate the 25th and 75th percentiles as determined by R software; whiskers extend to minimum and maximum values. RPMB, reads per million of bacterial reads.


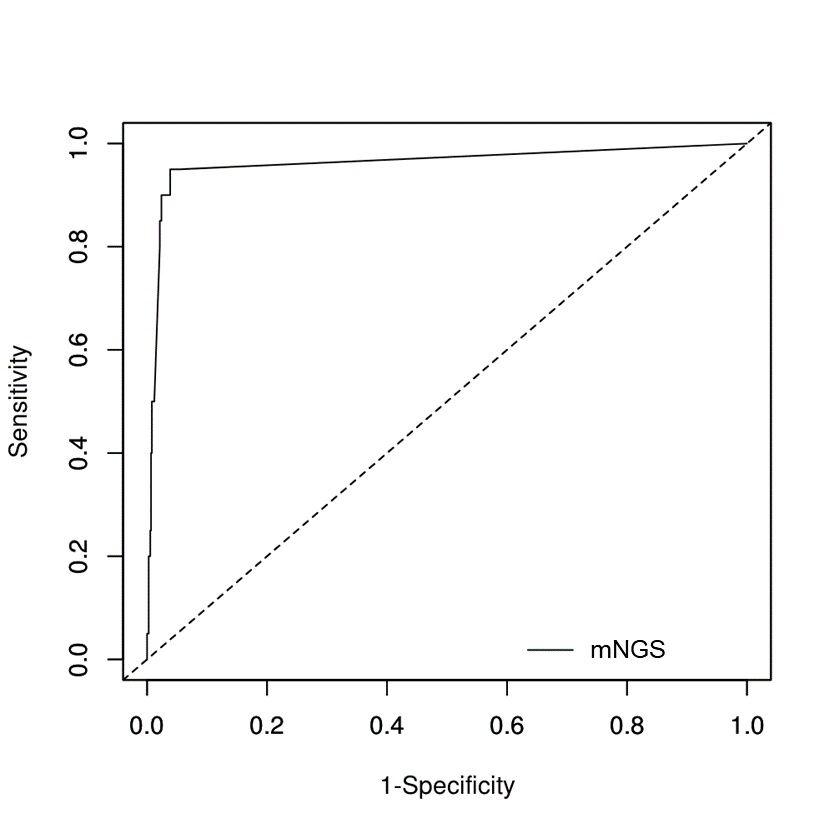


Additional figure 4: Receiver operating characteristics curve for determination of *MT* to differentiate concentration [GEq/mL] of SOI when present above/below clinical threshold in culture tests.

# Additional Tables

| **ID** | **Sample type** | **Bacterial species** | **Quantification (log10) ^a^** | | **RNsoi [RPMB] ^b^** | **16S MetaPhlAn2 markers** | **RNspc [RPMB] ^c^** |
| --- | --- | --- | --- | --- | --- | --- | --- |
|  |  |  | **Microbial culture** | **mNGS** |  |  |  |
| T01 | mini-BAL | *S. aureus* | **6** | **7,4** | 989770 | + | **884** |
| T02 | mini-BAL |  |  |  |  |  | **2442** |
| T03 | mini-BAL | *S. aureus* | **3** | **6,2** | 73040 | + | **1234** |
|  |  | *S. pneumoniae* | **3** | **4,9** | 2917 |  |  |
| T04 | mini-BAL | *C. freundii* | 2 | **4,3** | 721 |  | **521** |
|  |  | *K. oxytoca* |  | **7,3** | *958560* | + |  |
| T05 | mini-BAL |  |  |  |  |  | **331961** |
| T06 | mini-BAL |  |  |  |  |  | **612358** |
| T07 | mini-BAL | *S. aureus* |  | **4,0** | *57744* | + | **135763** |
| T08 | mini-BAL |  |  |  |  |  | **354289** |
| T09 | mini-BAL |  |  |  |  |  | **776411** |
| T10 | mini-BAL | *S. pneumoniae* |  | **3,9** | *17120* | + | **78257** |
| T11 | mini-BAL |  |  |  |  |  | **31116** |
| T12 | mini-BAL |  |  |  |  |  | **1321** |
| T13 | mini-BAL |  |  |  |  |  | **902605** |
| T14 | mini-BAL |  |  |  |  |  | **922099** |
| T15 | mini-BAL |  |  |  |  |  | **924367** |
| T16 | BAL | *C. freundii* |  | **6,1** | *239169* | + | **2391** |
|  |  | *E. coli* |  |  | *5212* | + |  |
|  |  | *K. pneumoniae* | 3 | **6,6** | 731024 | + |  |
| T17 | BAL | *K. pneumoniae* |  | **7,5** | *988952* | + | 422 |
|  |  | *S. aureus* | 2 | **5,1** | 2394 | + |  |
| T18 | BAL | *E. coli* | **>5** | **7,0** | 629675 | + | **775** |
|  |  | *S. aureus* | **>5** | **6,3** | 71078 | + |  |
| T19 | BAL | *S. aureus* | **5** | **6,3** | 863430 | + | **11046** |
| T20 | BAL | *S. pneumoniae* |  | **4,7** | *87286* | + | **51792** |
| T21 | BAL | *C. freundii* | 2 | **7,2** | 772664 | + | **637** |
|  |  | *E. coli* |  |  | *14038* | + |  |
|  |  | *K. pneumoniae* |  | **6,5** | *176247* | + |  |
| T22 | mini-BAL |  |  |  |  |  | **12226** |
| T23 | BAL |  |  |  |  |  | **141765** |
| T24 | mini-BAL |  |  |  |  |  | **5307** |
| T25 | mini-BAL | *H. influenzae* |  | **6,3** | *115715* | + | **2052** |
| T26 | BAL | *E. coli* | **>5** | **7,7** | 870427 | + | 211 |
|  |  | *P. mirabilis* | **>5** | **3,9** | 88 |  |  |
|  |  | *S. aureus* | **>5** | **5,6** | 3274 | + |  |
| T27 | BAL | *H. influenzae* | **>5** | **5,9** | 718264 | + | **36503** |
| T28 | BAL | *H. influenzae* | **>5** | **7,1** | 818867 | + | **2679** |
|  |  | *P. vulgaris* | **4** | **4,0** | 1593 |  |  |
|  |  | *S. aureus* | **5** | **5,0** | 10836 | + |  |
| T29 | BAL | *E. coli* |  | **5,4** | *23817* | + | **1209** |
|  |  | *P. aeruginosa* | **>5** | **6,9** | 876624 | + |  |
| T30 | BAL |  |  |  |  |  | **128034** |
| T31 | BAL | *H. influenzae* |  | **3,8** | *1055* | + | **5696** |
|  |  | *S. aureus* | **5** | **6,0** | 217608 | + |  |
| T32 | mini-BAL | *S. pneumoniae* |  | **4,2** | *39789* | + | **74542** |
| T33 | BAL | *S. pneumoniae* |  | 3,2 | *12522* | + | **247773** |
| T34 | BAL |  |  |  |  |  | **89686** |
| T35 | mini-BAL | *E. coli* | 2 | **6,6** | 35394 |  | 124 |
|  |  | *S. maltophilia* | **5** | **7,9** | 663154 | + |  |
| T36 | mini-BAL |  |  |  |  |  | **575708** |
| T37 | BAL |  |  |  |  |  | **15739** |
| T38 | mini-BAL |  |  |  |  |  | **569** |
| T39 | BAL | *K. pneumoniae* | **>5** | **7,6** | 949758 | + | 308 |
| T40 | BAL |  |  |  |  |  | **84757** |
| T41 | mini-BAL |  |  |  |  |  | **11089** |
| T42 | BAL |  |  |  |  |  | **3781** |
| T43 | BAL | *H. influenzae* |  | **5,7** | *4609* | + | 322 |
|  |  | *P. mirabilis* | **>5** | **7,7** | 959346 | + |  |
|  |  | *S. aureus* | 2 | 2,4 | 4 |  |  |
| T44 | BAL | *S. aureus* | **4** | **6,1** | 689401 | + | **11952** |
| T45 | BAL | *S. aureus* | **4** | **3,7** | 5875 | + | **26606** |

Additional table 1: Results of microbial culture, mNGS and qPCR detections on training set of samples. (a) Quantification results are provided in log10 of concentration, in CFU/mL for microbial culture and in GEq/mL for mNGS and qPCR. Values above clinical threshold for microbial culture (3 for mini-BAL and 4 for BAL) or above *MT* (3,72) for mNGS are shown in bold. (b) Values in italic were removed from the calculation of *DT_SOI_* because the negative culture result for a given SOI was associated with a substantial number of sequence reads assigned to this SOI, which was confirmed by the 16S/MetaPhlAn2 markers search. (c) Values above *DT_SPC_* (475 RPMB) are in bold. (+) is the detection of 16S/MetaPhlAn2 markers specific to the corresponding SOI.

# Additional references

1. Wood DE, Salzberg SL. Kraken: ultrafast metagenomic sequence classification using exact alignments. Genome Biol. 2014;15:R46. doi: <https://doi.org/10.1186/gb-2014-15-3-r46>.

2. Ye SH, Siddle KJ, Park DJ, Sabeti PC. Benchmarking Metagenomics Tools for Taxonomic Classification. Cell. 2019;178:779-94. doi: <https://doi.org/10.1016/j.cell.2019.07.010>.

3. Glassing A, Dowd SE, Galandiuk S, Davis B, Chiodini RJ. Inherent bacterial DNA contamination of extraction and sequencing reagents may affect interpretation of microbiota in low bacterial biomass samples. Gut Pathog. 2016;8. doi: <https://doi.org/10.1186/s13099-016-0103-7>.

4. Salter SJ, Cox MJ, Turek EM, Calus ST, Cookson WO, Moffatt MF, et al. Reagent and laboratory contamination can critically impact sequence-based microbiome analyses. BMC Biol. 2014;12. doi: <https://doi.org/10.1186/s12915-014-0087-z>.

5. Silkie SS, Tolcher MP, Nelson KL. Reagent decontamination to eliminate false-positives in Escherichia coli qPCR. J Microbiol Methods. 2008;72:275-82. doi: <https://dx.doi.org/10.1016/j.mimet.2007.12.011>.

6. O'Sullivan DM, Laver T, Temisak S, Redshaw N, Harris KA, Foy CA, et al. Assessing the accuracy of quantitative molecular microbial profiling. Int J Mol Sci. 2014;15:21476-91. doi: <https://doi.org/10.3390/ijms151121476>.

7. Goksuluk D, Korkmaz S, Zararsiz G, Karaağaoğlu A. easyROC: An Interactive Web-tool for ROC Curve Analysis Using R Language Environment. The R Journal. 2016;8:213-30. doi: <http://dx.doi.org/10.32614/RJ-2016-042>.

8. Youden WJ. Index for rating diagnostic tests. Cancer. 1950;3:32-5. doi: <https://dx.doi.org/10.1002/1097-0142(1950)3:1><32::aid-cncr2820030106>3.0.co;2-3.
